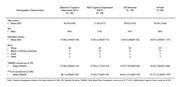# Brainstem Atrophy across the AD Clinical Spectrum: An Automated Volumetric MRI study

**DOI:** 10.1002/alz.084191

**Published:** 2025-01-09

**Authors:** Deepali M Bhalla, Barbara B. Bendlin, Piero G. Antuono, Elias Granadillo

**Affiliations:** ^1^ Medical College of Wisconsin, Milwaukee, WI USA; ^2^ University of Wisconsin‐Madison, Madison, WI USA

## Abstract

**Background:**

Studies suggest that structural changes in the midbrain are linked to Alzheimer’s disease (AD) symptoms such as memory, sleep, and emotional disturbances. Brainstem atrophy, particularly in the locus coeruleus, has also been linked with poorer executive function. Studies showing brainstem as one of the first regions affected by AD largely employed novel imaging techniques such as neuromelanin‐sensitive MRI, which is limited to research environments. Prior studies have established the utility of hippocampal measures using automated volumetric software such as Neuroreader (NR) aiding in diagnosis of AD, and is emerging as an inexpensive and easily accessible tool. Here, we used NR to assess brainstem (BS) volumes of AD patients to examine the potential to capture an effect using conventional T1‐weighted MRI.

**Methods:**

We conducted a retrospective chart review (N=300) of participants who underwent volumetric scan evaluated with NR after 2015 (Table 1). NR was used to generated BS and hippocampal volumes corrected for total intracranial volume (BS‐Vc and Hip‐Vc) among 3 groups (N=100 each): subjective cognitive impairment (SCI), mild cognitive impairment (MCI), and AD Dementia. Categorization of participants was based on the current clinical diagnostic criteria for AD clinical syndrome. Multiple regression models were run to determine group‐wise differences in BS‐Vc and Hip‐Vc, correcting for age, gender, and education.

**Results:**

Preliminary results showed no difference in BS‐Vc across groups. Hip‐Vc was significantly different between SCI and AD (β = ‐0.048, p <0.001) and MCI and AD (β = ‐0.033, p =0.001) groups. Interestingly, with correction for age, education level, and disease stage, male gender was associated with lower BS‐Vc (β = ‐0.053, p <0.001); consistent with AD literature suggesting a differential susceptibility to AD pathology based on gender.

**Conclusion:**

We found no differences in BS‐Vc across the AD clinical spectrum, possibly due to low power of this study, or limitations in NR to detect atrophy in brainstem sub‐structures such as the locus coeruleus. Differences in BS‐Vc across genders was independent of disease status, suggesting a role of gender susceptibility in AD that is specific to the brainstem. Further exploration of these findings in future studies with increased power is needed.